# Clinical significance of peripheral blood-derived inflammation markers in advanced gastric cancer after radical resection

**DOI:** 10.1186/s12893-020-00884-8

**Published:** 2020-10-02

**Authors:** Lihu Gu, Mian Wang, Xuena Cui, Jiahang Mo, Lingling Yuan, Feiyan Mao, Kang Zhang, Derry Minyao Ng, Ping Chen, Dongjie Wang

**Affiliations:** 1 Department of General Surgery, HwaMei Hospital, University of Chinese Academy of Sciences, Ningbo, Zhejiang China; 2 Key Laboratory of Diagnosis and Treatment of Digestive System Tumors of Zhejiang Province, Ningbo, Zhejiang China; 3 Ningbo Clinical Research Center for Digestive System Tumors, Ningbo, Zhejiang China; 4 Infection Department, Ningbo Yinzhou No. 2 Hospital, Ningbo, Zhejiang China; 5 Intensive Care Unit, Ningbo Yinzhou No. 2 Hospital, Ningbo, Zhejiang China; 6 The Second Clinical Medical College, Zhejiang Chinese Medical University, Hangzhou, Zhejiang China; 7 Medical College of Ningbo University, Ningbo, Zhejiang China; 8 Department of Clinical Laboratory, HwaMei Hospital, University of Chinese Academy of Sciences, Northwest Street 41, Haishu District, Ningbo, 315010 Zhejiang China

**Keywords:** Gastric cancer, Systemic inflammatory marker, Platelet lymphocyte ratio, Prognosis, Chemotherapy

## Abstract

**Background:**

The prognostic significance of peripheral blood-derived inflammation markers in patients with gastric cancer (GC) has not been elucidated. This study aimed to investigate the relationship between systemic inflammatory markers and GC prognosis.

**Methods:**

A prospective observational cohort study involving 598 patients was conducted to analyze the prognosis of GC based on systemic inflammatory markers. The following peripheral blood-derived inflammation markers were evaluated: the neutrophil-lymphocyte ratio (NLR), platelet lymphocyte ratio (PLR), systemic immune-inflammation index (SII), C-reactive protein/albumin (CRP/Alb) ratio, Glasgow Prognostic Score (GPS), modified Glasgow Prognostic Score (mGPS), prognostic nutrition index (PNI), and prognostic index (PI). The receiver operating characteristics (ROC) curve and the Youden index were used to determine the optimal cutoff values. Univariate and multivariate analysis of prognostic factors was conducted accordingly.

**Results:**

The optimal cutoff values of the PNI, fibrinogen, NLR, PLR, SII, and CRP/Alb were 49.5, 397 ng/dl, 2.5, 154, 556, and 0.05, respectively. Multivariate analysis showed that age, PLR, TNM stage, and chemotherapy were the independent prognostic factors for advanced gastric cancer (AGC). Adjuvant chemotherapy improved the long-term prognosis of patients with PLR ≥154, but chemotherapy had no significant effect on the survival of patients with PLR < 154.

**Conclusions:**

Our findings show that higher PLR (≥154) is an independent risk factor for poor prognosis in GC patients. Besides, PLR can predict adjuvant chemotherapy (oxaliplatin/5-fluorouracil combination) response in patients with GC after surgery.

## Background

Gastric cancer (GC) is one of the most common malignant tumors and that poses a serious threat to human health, especially in Asia. Approximately 300,000 deaths and 400,000 new cases of GC occur in China every year [1]. Despite the advancement in diagnostic and treatment methods, the prognosis of advanced gastric cancer (AGC) patients has remained poor [2]. Tumor, Node, Metastasis (TNM) staging based on the International Union Against Cancer (UICC)/American Joint Committee on Cancer (AJCC) guidelines is currently the standard approach of determining the prognosis of GC patients [3]. However, several prognostic factors related to GC have been proposed, which include peripheral blood-derived inflammation markers, such as neutrophil-lymphocyte ratio (NLR), platelet lymphocyte ratio (PLR), systemic immune-inflammation index (SII), C-reactive protein/albumin (CRP/Alb) ratio, and Glasgow Prognostic Score (GPS) [4–6].

Some studies have combined the TNM staging system with GC related risk factors to improve the accuracy of the long-term prognosis of the disease [7]. Routine peripheral blood-derived inflammation markers are closely associated with the pathogenesis of GC [8]. Moreover, the use of these markers as prognostic factors is advantageous because most of the peripheral blood-derived inflammation markers belong to the routine test items; the test cost is cheap and does not require special equipment.

Park indicated that preoperative body mass index (BMI) and prognostic nutritional index (PNI), as well as their postoperative changes, are related to the prognosis of stage II/III GC [9]. Also, Jagadesham reported that the combination of one or more markers of systemic inflammation could precisely predict the prognosis of advanced adenocarcinoma of the esophagus [10]. Studies have suggested that combining NLR and PLR could significantly improve the accuracy of predicting the first-line chemosensitivity in AGC [11]. Contrarily, Xu et al. indicated that CRP/Alb might be a promising predictor of early recurrence (recurrence within 12 months after radical gastrectomy) and postoperative chemotherapy in stage III GC [12].

Unfortunately, most of these findings are based on small sample retrospective studies with insufficient evidence, which could be the reason for the inconsistencies among the various reports. Herein, we designed a prospective observational cohort to examine the relationship between peripheral blood-derived inflammation markers and the prognosis of GC. Also, we hypothesized that derangements in one or more systemic inflammation markers may be associated with poor disease outcomes and the ineffectiveness of chemotherapy.

## Methods

### Study population

This was a prospective observational cohort study involving patients who underwent radical gastrectomy from January 2013 to December 2016 at HwaMei Hospital, University of Chinese Academy of Sciences, and was approved by the Ethics Committee of the HwaMei Hospital and the University of Chinese Academy of Sciences (approval NO. PJ-NBEY-KY-2019-153-01). Written consent was obtained from all patients before enrollment. The inclusion criteria are as follows: (1) patients with histologically proven primary adenocarcinoma of the stomach; (2) patients without a history of gastrectomy or any other malignant tumor; (3) patients negative for any inflammatory or hematological diseases; (4) patients with pathologically negative resection margins (R0, 5) patients not receiving any neoadjuvant chemoradiotherapy; (6) follow-up period of at least 36 months. A treatment plan that includes a 5-fluorouracil (5-FU)-based or any platinum-based adjuvant chemotherapy is recommended for all patients diagnosed with stage II-III of the disease [13].

### Systemic inflammatory markers and histological examination

The whole blood and clinicopathological data were obtained 1 week before initial treatment. Blood samples were collected for routine laboratory tests, which included complete blood count, CRP, albumin, fibrinogen, and tumor markers, such as carcinoembryonic antigen (CEA). The following common peripheral blood-derived inflammation markers were included based on previous studies: NLR, PLR, SII, CRP/Alb, GPS, modified Glasgow Prognostic Score (mGPS), PNI, and prognostic index (PI).

All surgical resection specimens were assessed according to the handling guideline of the third edition of the Japanese classification of gastric carcinoma. And the staging was conducted by pathologists using the 8th edition of the UICC/AJCC TNM staging system [3].

### Follow-up

Patients included in the study were followed up with every 3–6 months for the first 2 years and then annually after until death or at least 5 years after the surgery. Disease-free survival (DFS) was defined as the time from surgery to death or until the patient is found to have either loco-regional recurrence or distant recurrence. Disease-specific survival (DSS) was defined as the time from surgery to death as a result of GC. Patients with no records of the above events were censored at the date of their last known contact. The median follow-up period for the entire cohort was 50 months (range 4–83 months), and all patient follow-up was stopped in December 2019.

### Statistical analysis

The continuous variables in the study were analyzed using either the independent sample *t-*test or Wilcoxon rank-sum test, while the categorical variables were analyzed using either the Pearson’s chi-squared test or Fisher’s exact test where applicable. The receiver operating characteristics (ROC) curve was calculated, and the Youden index (maximum = sensitivity + specificity - 1) was used to determine the optimal cutoff value for the number of lymph nodes retrieved, PNI, fibrinogen, NLR, PLR, SII, and CRP/Alb. Any potentially relevant factors derived from the univariate analysis were assessed in the multivariate model using Cox’s regression. We also calculated the hazard ratios (HR) and 95% confidence intervals (CI). The DFS and DSS rate were obtained using the Kaplan-Meier method, and the log-rank test was used to determine if the result was statistically significant. A *p* < 0.05 was considered statistically significant, and all statistical tests were performed 2-sided. SPSS software (version 25.0, SPSS Inc. IL, USA) was used for all analyses.

## Results

A total of 598 patients were recruited from January 2013 to December 2016. The 5-year DFS and DSS rates of all patients were 72.6 and 75.4%, respectively. Male patients were about twice as many as female patients, and the tumors in the distal stomach accounted for 77% of all the tumors. Concerning the GC staging, patients with GC stage I, II, and III were 119, 113, and 366, respectively. The 5-year DFS and DSS rates after surgery for stage I patients was 97 and 98%, respectively, whereas, for stage II patients, the rates were 81.4 and 85.8%, respectively. For stage III patients, the rates were 52.1 and 55.6%, respectively (Supplementary Table 1).

A total of 376 patients received adjuvant chemotherapy, of which 239 received SOX regimen [14] and 112 XELOX [15]. The remaining 25 patients received other chemotherapy treatments [16].

### Optimal cutoff analysis

The optimal cutoff value of the number of lymph nodes retrieved, PNI, fibrinogen, NLR, PLR, SII, and CRP/Alb were 30, 49.5, 397 ng/dl, 2.5, 154, 556, and 0.05, respectively (Supplementary Table 2).

### Clinicopathological factors and survival analysis

A total of 23 potential risk factors were selected, and from the univariate analysis results, we found 17 clinicopathological characteristics that were significantly associated with the 5-year DFS rate in all enrolled patients, the clinicopathological factors are as follows: age, tumor location, type of gastrectomy, tumor size, perineural invasion, lymphovascular invasion, T stage, N stage, chemotherapy, PNI, fibrinogen, NLR, PLR, SII, GPS, CRP/Alb, and CEA (Table 1). The multivariate Cox proportional hazards model analysis determined that age, T stage, N stage, number of lymph nodes retrieved, and PLR were independent prognostic factors for GC (Table 1).

**Table 1:** Univariate and multivariate analysis of prognostic factors in patients with gastric cancer

Clinicopathological feature	Univariate analysis			Multivariate analysis		
	HR	95% CI	*p* value	HR	95% CI	*p* value
Age (years)						
≤ 60	1			1		
> 60	1.62	1.16–2.25	**0.005**	1.63	1.15–2.31	**0.007**
Gender						
Male	1					
Female	1.01	0.73–1.40	0.967			
BMI						
≤ 24 kg/m^2^	1					
> 24 kg/m^2^	0.77	0.52–1.13	0.175			
Tumor location						
Upper third	1					
Middle third	0.53	0.29–0.98	**0.041**			
Lower third	0.47	0.31–0.71	**< 0.001**			
Two-thirds or more	0.76	0.27–2.15	0.600			
Gastrectomy						
Distal	1					
Total	1.57	1.10–2.23	**0.013**			
Proximal	NA					
Tumor size						
≤ 5 cm	1					
> 5 cm	2.60	1.90–3.54	**< 0.001**			
Histologic type						
Differentiated	1					
Undifferentiated	1.29	0.95–1.76	0.106			
Perineural invasion						
Absence	1					
Presence	2.73	2.00–3.72	**< 0.001**			
Lymphovascular invasion						
Absence	1					
Presence	2.78	2.02–3.83	**< 0.001**			
T category						
T1	1			1		
T2	4.30	1.56–11.83	**0.005**	3.04	1.08–8.57	**0.036**
T3	2.04	0.25–16.92	0.510	1.69	0.20–14.50	0.633
T4a	15.79	6.97–35.76	**< 0.001**	8.22	3.26–20.75	**< 0.001**
T4b	28.21	9.78–81.36	**< 0.001**	13.05	3.98–42.82	**< 0.001**
N category						
N0	1			1		
N1	3.22	1.76–5.90	**< 0.001**	1.70	0.88–3.26	0.112
N2	4.71	2.75–8.05	**< 0.001**	2.09	1.15–3.81	**0.016**
N3a	11.35	6.82–18.89	**< 0.001**	5.38	3.00–9.65	**< 0.001**
N3b	24.19	13.27–44.07	**< 0.001**	11.01	5.50–22.04	**< 0.001**
Chemotherapy						
No	1			1		
Yes	1.58	1.12–2.22	**0.009**	0.70	0.48–1.01	0.059
Number of lymph nodes retrieved						
≤ 15	1			1		
16–29	0.75	0.50–1.13	0.169	0.46	0.30–0.70	**< 0.001**
≥ 30	1.36	0.88–2.11	0.166	0.52	0.32–0.84	**0.008**
PI						
0	1					
1	1.54	0.96–2.49	0.075			
PNI						
< 49.5	1					
≥ 49.5	0.40	0.27–0.59	**< 0.001**			
Fibrinogen						
< 397 ng/dl	1					
≥ 397 ng/dl	2.06	1.51–2.81	**< 0.001**			
NLR						
< 2.5	1			1		
≥ 2.5	1.69	1.24–2.31	**0.001**	0.70	0.48–1.01	0.059
PLR						
< 154	1			1		
≥ 154	2.21	1.62–3.01	**< 0.001**	1.70	1.20–2.42	**0.003**
SII						
< 556	1					
≥ 556	1.94	1.43–2.64	**< 0.001**			
GPS						
0	1					
1	1.76	1.24–2.49	**0.002**			
2	1.89	1.04–3.43	**0.037**			
mGPS						
0	1					
1	1.53	0.68–3.46	0.307			
2	1.66	0.92–3.00	0.091			
CRP/Alb						
< 0.05	1					
≥ 0.05	1.91	1.40–2.60	**< 0.001**			
CEA						
≤ 5 ng/mL	1					
> 5 ng/mL	1.76	1.16–2.68	**0.008**			

*HR* Hazard ratios, *CI* Confidence interval, *BMI* Body mass index, *PNI* Prognostic nutritional index, *PI* Prognostic index, *GPS* Glasgow Prognostic Score, *mGPS* Modified Glasgow Prognostic Score, *NLR* Neutrophil-lymphocyte ratio, *PLR* Platelet-lymphocyte ratio, *CRP/Alb* C-reactive protein/albumin, *SII* Systemic immune-inflammatory index

### Risk factors for advanced gastric cancer

As there is a close correlation between the TNM stage and the 5-year DFS, we hypothesis that the number of lymph nodes extracted may be associated with the prognosis. If the total number of Lymph nodes extracted is less than 16, it would be defined as inadequate lymph node dissection, which is especially important for AGC. Therefore, only patients with the AGC lymph nodes ≥16 were included in the analysis. Multivariate analysis showed that age, PLR, and TNM stages were associated with 5-year DFS. Also, age, PLR, TNM stage, and chemotherapy were associated with 5-year DSS (Table 2).

**Table 2:** Multivariate analysis of prognostic factors in patients with advanced gastric cancer

Clinicopathological feature	Multivariate analysis DFS			Multivariate analysis DSS		
	HR	95% CI	*p* value	HR	95% CI	*p* value
Age (years)						
≤ 60	1			1		
> 60	1.61	1.10–2.35	**0.014**	1.61	1.06–2.42	**0.024**
PLR						
< 154	1			1		
≥ 154	2.00	1.40–2.84	**< 0.001**	2.09	1.43–3.04	**< 0.001**
TNM						
II	1			1		
III	4.46	2.51–7.91	**< 0.001**	5.94	3.01–11.74	**< 0.001**
Chemotherapy						
No	1			1		
Yes	0.70	0.48–1.04	0.074	0.66	0.45–0.99	**0.043**

*DFS* Disease-free survival, *DSS* Disease-specific survival, *HR* Hazard ratios, *CI* Confidence interval, *PLR* Platelet-lymphocyte ratio

### Risk factors associated with stage II and III gastric cancer

**Fig. 1:**
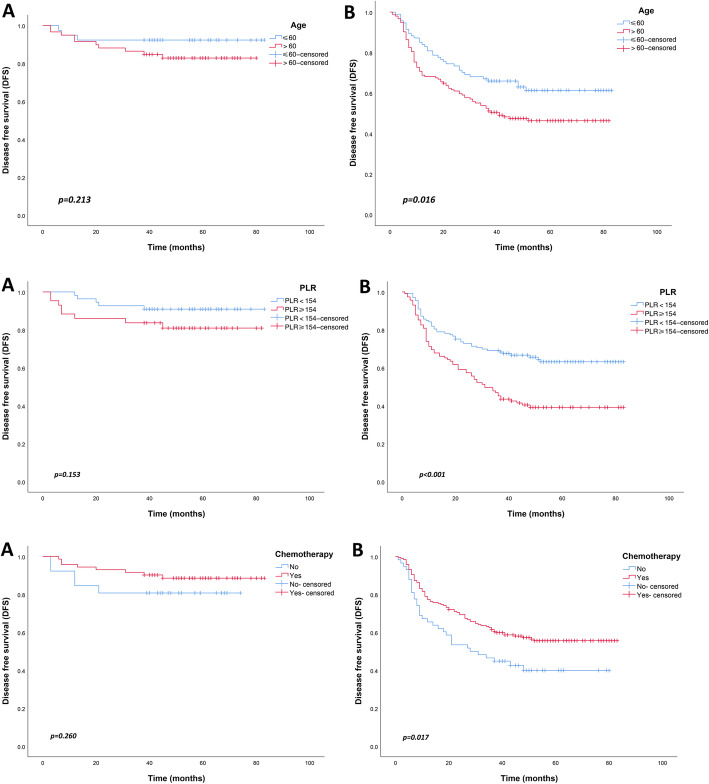
Disease-free survival (DFS) of patients with advanced gastric cancer according to the risk factors. (**a**) stage II; (**b**) stage III

The Kaplan-Meier curves were used to determine the long-term disease outcomes of GC patients in stage II and stage III. In GC stage II, the 5-year DFS rates were similar among patients with age ≤ 60 and age > 60 (*p* = 0.213). However, in stage III, the 5-year DFS rate of GC patients aged > 60 was worse than those aged ≤60 (*p* = 0.016). Similarly, in GC stage III, the 5-year DFS rate of patients with PLR ≥154 was worse than those with PLR < 154 (*p* < 0.001). But this phenomenon was not observed in GC stage II (*p* = 0.153). The effect of chemotherapy on the prognosis of patients with stage II GC was not statistically significant (*p* = 0.260). In contrast, chemotherapy significantly improved the 5-year DFS in patients with stage III GC (*p* = 0.017) (Fig. 1).

**Fig. 2:**
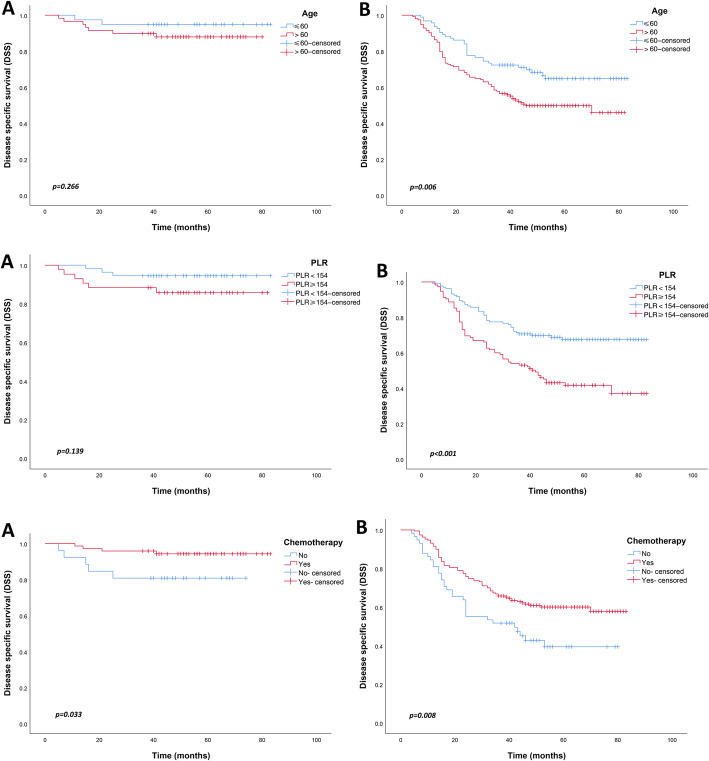
Disease-specific survival (DSS) of patients with advanced gastric cancer according to the risk factors. (**a**) stage II; (**b**) stage III

With the 5-year DSS as a long-term prognostic index, age, PLR, and chemotherapy had the same effect on the prognosis of stage II and III GC patients as 5-year DFS, except that chemotherapy also improved the 5-year DSS of stage II GC patients (*p* = 0.033) (Fig. 2).

**Fig. 3:**
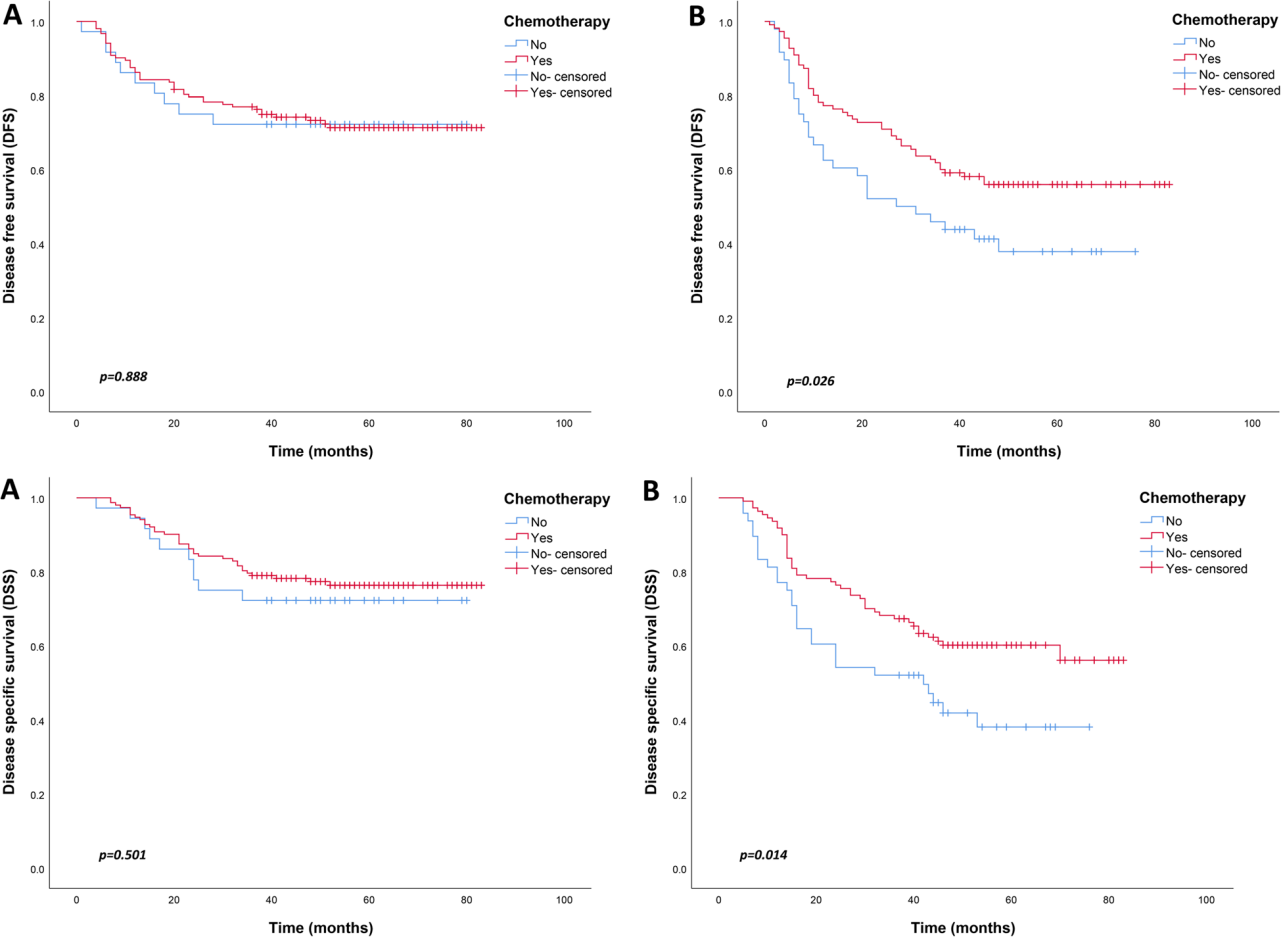
Comparison of survival curves between patients with or without chemotherapy in advanced gastric cancer. (**a**) PLR < 154; (**b**) PLR ≥154

When considered together with the above observations, PLR was found to be an independent risk factor for the prognosis of AGC. Patients with AGC were divided into either the PLR < 154 and PLR ≥154 subgroups. Further analysis of the clinicopathological factors between the two groups revealed that tumor lesions in the PLR < 154 group were smaller than those in the PLR ≥154 group. But there was no other difference between the groups (Table 3). Further analysis of the relationship between chemotherapy and PLR showed that chemotherapy improved the long-term prognosis of patients in the PLR ≥154 group, including 5-year DFS and DSS (*p* = 0.026, *p* = 0.014, respectively). Notably, chemotherapy had no significant effect on the long-term prognosis of patients in the PLR < 154 group (Fig. 3).

**Table 3:** Association between PLR and the patients’ characteristics

	PLR < 154	PLR ≥ 154	*p* value
Age (year)			0.223
≤ 60	78	55	
> 60	110	103	
Gender			0.818
Male	128	105	
Female	60	53	
Tumor location			0.941
Upper third	26	47	
Middle third	23	17	
Lower third	133	116	
Two-thirds or more	6	4	
Tumor size			**< 0.001**
≤ 5 cm	135	73	
> 5 cm	53	85	
Histologic type			0.156
Differentiated	74	75	
Undifferentiated	114	83	
Perineural invasion			0.194
Absence	79	78	
Presence	109	80	
Lymphovascular invasion			0.509
Absence	77	59	
Presence	111	99	
T category			0.835
T1	7	4	
T2	15	10	
T3	7	6	
T4a	153	135	
T4b	6	3	
N category			0.416
N0	35	28	
N1	39	29	
N2	58	41	
N3a	45	43	
N3b	11	17	
CEA			0.275
≤ 5 ng/mL	166	133	
> 5 ng/mL	22	25	

*PLR* Platelet-lymphocyte ratio

## Discussion

Many studies have been conducted to investigate the correlation between peripheral blood-derived inflammation markers and tumor prognosis [17, 18]. Liu showed that CRP/Alb was an independent prognostic marker for patients with ovarian cancer [19]. Also, the NLR and PLR are prognostic factors in patients with non-small cell lung cancer after stereotactic radiation therapy [20]. The independent risk factors for poor GC prognosis include NLR, PLR, fibrinogen, PNI, GPS, CRP/Alb, among others. Also, some studies have combined these systemic inflammatory markers with or without TNM stage to provide new prognostic tools [21–23]. However, most of these studies were retrospective, and reported inconsistent results, particularly on the significance of each inflammatory index and the threshold value.

Therefore, in addition to the peripheral blood-derived inflammation markers reported in previous studies, this study further explored the prognostic value of some conventional systemic inflammatory marker in patients with GC. We prospectively analyzed 598 GC patients after radical surgery and found that independent risk factors for poor prognosis of GC included age, T stage, N stage, number of lymph nodes retrieved, and PLR. Currently, TNM staging is the standard prognostic tool for GC. Given the excellent prognosis of early gastric cancer, our focus was to analyze the prognosis of patients with AGC (stage II/III). Many studies have demonstrated that the number of lymph node dissection has an impact on the prognosis of GC; therefore, we further excluded patients with an inadequate amount of lymph node dissection (less than [16, 24–26]).

Independent risk factors for AGC included age, PLR, TNM stages, and chemotherapy. Previous studies have investigated the effect of age on GC prognosis; however, most of these studies found no significant association between age and GC prognosis [27–29]. Takatsu analyzed 5000 GC cases and found that early-onset GC (age < 40 years) was likely to present lymph node metastases. But the survival rate of young GC patients was equivalent to that of older GC patients (age ≥ 60 years) [30]. In the present study, older GC patients (age > 60 years) had a worse prognosis, which was closely related to tumor recurrence.

Nutritional status is associated with survival in patients with malignant tumors, including GC. Preoperative underweight and low PNI are considered poor prognostic factors. Park suggested that careful nutritional intervention after surgery could improve the survival rate [9]. Besides, a meta-analysis concluded that a low PNI is significantly associated with poor overall survival except for stage IV GC [31]. However, consistent with the results of Li et al. [32], we observed that PNI was not associated with prognosis. Also, our results did not show a correlation between fibrinogen and the prognosis of GC. Recent studies have shown that fibrinogen is one of the risk factors for poor prognosis in upper gastric cancer [33]. Fibrinogen is the primary acute-phase protein, and as a critical component of the hemostatic system, it regulates the systemic inflammatory state and cancer progression. However, its clinical significance in the prognosis of GC has not been elucidated.

The NLR and PLR are the most extensively studied markers of peripheral blood-derived inflammation, which are associated with the prognosis of GC. Accumulating evidence has shown that NLR and PLR are associated with distant metastases during GC progression [34, 35]. Kim reported that although both the PLR and NLR could reflect the prognosis, the NLR was more predictive of overall survival than the PLR in GC [36]. Also, they suggested that NLR and PLR might be associated with lymph node metastasis in early gastric cancer [37]. On the contrary, Zhu et al. indicated that NLR and PLR could not predict lymph node metastasis and prognosis in early gastric cancer [38]. In the present study, PLR was significantly correlated with the prognosis of GC, but there was no statistical difference between NLR and prognosis of GC. This observation seems to be inconsistent with previous studies, but the exact mechanism is still unclear. However, the inconsistencies could be because most previous studies focused on overall survival as the primary outcome, whereas herein, we used tumor recurrence and tumor-related mortality as observational indicators, which seem to be more accurate. In addition, the clinicopathological characteristics were similar between the PLR elevating group (PLR < 154) and the PLR decreasing group (PLR ≥154), except for tumor size, which further suggested that PLR might influence the prognosis of tumor through other mechanisms. A recent meta-analysis has revealed that PLR is associated with prognosis of GC [39].

Abnormal levels of CRP and Alb have been related to poor prognosis of tumor patients. It is noteworthy that the combinations of these two indicators can enhance the accuracy to predict the recurrence of multiple tumors. Among them, the most common evaluation criteria after combination include GPS and CRP/Alb. Besides, many studies have used GPS to predict the prognosis of various tumors, including GC. Hsueh recently recommended the use of GPS as a predictive and prognostic factor in patients with GC. A significant correlation was observed between the GPS, short-term postoperative complications, and long-term survival outcomes in patients with GC undergoing D2 gastrectomy [40]. Some studies have indicated that GPS and mGPS, used either alone or in combination, represent an independent prognostic factor for long-term outcome in resected GC [41, 42]. However, Walsh’s results did not show a correlation between prognosis of patients and mGPS levels, although mGPS was associated with advanced GC stage [17]. Liu et al. retrospectively analyzed 455 patients with resectable GC and showed that CRP/Alb, rather than GPS and mGPS, was associated with overall survival [43]. Similarly, Xu [12] and Lu [44] et al. also observed that CRP/Alb and CRP/prealbumin were associated with recurrence of GC based on the data from a phase III randomized clinical trial. On the contrary, our results showed that although CRP/Alb and GPS were associated with prognosis in the univariate analysis, the multivariate analysis showed that both were not related to long-term survival.

Consistent with the results of previous clinical trials, our findings showed that chemotherapy could significantly improve the prognosis of AGC, especially for patients with stage III GC [45, 46]. Moreover, many studies have investigated the correlation between peripheral blood-derived inflammation markers and the effects of chemotherapy, to guide the selection of chemotherapy-sensitive patients. A study suggested that the sensitivity of chemotherapy (oxaliplatin/5-fluorouracil combination) might be closely related to NLR, PLR, and their changes in metastatic gastric cancer [47]. Hirahara believed that the combination of NLR and PLR might be more effective in predicting the chemotherapy response in patients with metastatic gastric cancer [48]. Besides, Tang [49] and Chen [50] concluded that PLR could predict the efficacy of neoadjuvant chemotherapy of GC patients treated with oxaliplatin and capecitabine regimens. In the current study, PLR effectively predicted adjuvant chemotherapy (oxaliplatin/5-fluorouracil combination) response in patients with AGC after surgery. For patients with PLR ≥154, chemotherapy significantly improved long-term survival, including DFS and DSS; however, patients with PLR < 154 did not benefit from adjuvant chemotherapy. Thus, we recommend that AGC patients with PLR ≥154 should actively receive adjuvant chemotherapy (oxaliplatin/5-fluorouracil combination) after surgery, whereas patients with PLR < 154 need to be cautious when choosing adjuvant chemotherapy.

This study systematically evaluated the relationship between peripheral blood-derived inflammation markers and the prognosis of GC. Notably, the potential ability of inflammatory markers to predict the effects of chemotherapy was further demonstrated. However, this study had a few limitations. Importantly, this was an observational study and was therefore influenced by other confounding factors. For example, lymphocytes, neutrophils, CRP and others may be affected by complications such as chronic bronchitis, pneumonia. The time of collection of blood samples is not fixed, which may affect the test results. Also, the sample size was relatively small, and this may have reduced the reliability of the findings. Thus, these results need to be further validated by large multicenter randomized clinical trials.

## Conclusion

Our findings show that PLR is significantly correlated with the prognosis of GC, especially for stage III. That is, higher PLR (≥154) is an independent risk factor for poor long-term survival rate of GC patients. Moreover, PLR can be used to predict adjuvant chemotherapy (oxaliplatin/5-fluorouracil combination) response in patients with GC after surgery.

## Supplementary information

**Additional file 1: Table S1.** Baseline clinicopathological characteristics.

**Additional file 2: Table S2.** Optimal cutoff analysis.

## Data Availability

We state that all the raw data for this study have been provided.

## Abbreviation

GC: Gastric cancer
AGC: Advanced gastric cancer
UICC: International Union Against Cancer
AJCC: American Joint Committee on Cancer
NLR: Neutrophil-lymphocyte ratio
PLR: Platelet lymphocyte ratio
SII: Systemic immune-inflammation index
CRP/Alb: C-reactive protein/albumin ratio
GPS: Glasgow Prognostic Score
mGPS: Modified Glasgow Prognostic Score
BMI: Body mass index
PNI: Prognostic nutritional index
PI: Prognostic index
DFS: Disease-free survival
DSS: Disease-specific survival
ROC: Receiver operating characteristics
HR: Hazard ratios
CI: Confidence intervals
