# Supplementary material for: Clinical significance of peripheral blood-derived inflammation markers in advanced gastric cancer after radical resection
Source: BMC Surg. 2020 Oct 2;20:219. doi: 10.1186/s12893-020-00884-8 (PMC7532590; doi:10.1186/s12893-020-00884-8)
Supplement: Supplementary file 1 — Additional file 1: Table S1. Baseline clinicopathological characteristics. [file 12893_2020_884_MOESM1_ESM.docx]

**Supplementary Table 1.** Baseline clinicopathological characteristics

|  | Number | % |  | Number | % |  | Number | % |
| --- | --- | --- | --- | --- | --- | --- | --- | --- |
| Age (mean ± SD; years) | 63.2±10.2 | | Histologic type | | | PI | | |
| Gender | | | Differentiated | 293 | 49.0 | 0 | 545 | 91.1 |
| Male | 404 | 67.6 | Undifferentiated | 305 | 51.0 | 1 | 53 | 8.9 |
| Female | 194 | 32.4 | T category | | | 2 | 0 | 0 |
| BMI (mean ± SD; kg/m^2^) | 22.1±3.4 | | T1 | 175 | 29.3 | GPS | | |
| Tumor location | | | T2 | 70 | 11.7 | 0 | 448 | 74.9 |
| Upper third | 63 | 10.5 | T3 | 14 | 2.3 | 1 | 117 | 19.6 |
| Middle third | 64 | 10.7 | T4a | 327 | 54.7 | 2 | 33 | 5.5 |
| Lower third | 460 | 76.9 | T4b | 12 | 2.0 | mGPS | | |
| Two-thirds or more | 11 | 1.8 | N category | | | 0 | 549 | 91.8 |
| Gastrectomy | | | N0 | 255 | 42.6 | 1 | 16 | 2.7 |
| Distal | 481 | 80.4 | N1 | 95 | 15.9 | 2 | 33 | 5.5 |
| Total | 115 | 19.2 | N2 | 122 | 20.4 | NLR (mean ± SD) | 2.9±2.4 | |
| Proximal | 2 | 0.3 | N3a | 98 | 16.4 | Fibrinogen (mg/dl; mean ± SD) | 401.0±106.1 | |
| Tumor size ( mean ± SD; cm) | 4.2±2.4 | | N3b | 28 | 4.7 | PLR (mean ± SD) | 159.8±92.6 | |
| Perineural invasion | | | Chemotherapy | | | CRP/Alb (mean ± SD) | 0.1±0.3 | |
| Absence | 372 | 62.2 | No | 222 | 37.1 | SII (mean ± SD) | 648.5±580.1 | |
| Presence | 226 | 37.8 | Yes | 376 | 62.9 | CEA | | |
| Lymphovascular invasion | | | Number of lymph nodes retrieved (range) | 22.0±9.3 | | ≤5 ng/mL | 536 | 89.6 |
| Absence | 337 | 56.4 | PNI (mean ± SD) | 46.6±6.7 | | >5 ng/mL | 62 | 10.4 |
| Presence | 261 | 43.6 |  |  |  |  |  |  |

BMI, Body mass index; PNI, Prognostic nutritional index; PI, Prognostic index; GPS, Glasgow Prognostic Score; mGPS, Modified Glasgow Prognostic Score; NLR, Neutrophil-lymphocyte ratio; PLR, Platelet-lymphocyte ratio; CRP/Alb, C-reactive protein/albumin; SII, Systemic immune-inflammatory index
