# Supplementary material for: Clinical significance of peripheral blood-derived inflammation markers in advanced gastric cancer after radical resection
Source: BMC Surg. 2020 Oct 2;20:219. doi: 10.1186/s12893-020-00884-8 (PMC7532590; doi:10.1186/s12893-020-00884-8)
Supplement: Supplementary file 2 — Additional file 2: Table S2. Optimal cutoff analysis. [file 12893_2020_884_MOESM2_ESM.docx]

| Markers | Cut-off points | Sensitivity | Specificity |
| --- | --- | --- | --- |
| Lymph nodes retrieved | 30 | 0.395 | 0.733 |
| PNI | 49.5 | 0.202 | 0.631 |
| fibrinogen | 397 ng/dl | 0.574 | 0.493 |
| NLR | 2.5 | 0.589 | 0.484 |
| PLR | 154 | 0.597 | 0.627 |
| SII | 556 | 0.589 | 0.530 |
| CRP/Alb | 0.05 | 0.550 | 0.562 |

**Supplementary Table 2.** Optimal cutoff analysis
